# A consensus genetic map of sorghum that integrates multiple component maps and high-throughput Diversity Array Technology (DArT) markers

**DOI:** 10.1186/1471-2229-9-13

**Published:** 2009-01-26

**Authors:** Emma S Mace, Jean-Francois Rami, Sophie Bouchet, Patricia E Klein, Robert R Klein, Andrzej Kilian, Peter Wenzl, Ling Xia, Kirsten Halloran, David R Jordan

**Affiliations:** 1 The Department of Primary Industries & Fisheries, Queensland (DPI&F), Hermitage Research Station, Warwick, QLD 4370, Australia; 2 CIRAD UMR DAP, TA A-96/03, Av Agropolis, 34398 Montpellier CEDEX 5, France; 3 Department of Horticulture and Institute for Plant Genomics and Biotechnology, Texas A&M University, College Station, TX 77843-2123, USA; 4 USDA-ARS, Southern Plains Agricultural Research Center, College Station, TX 77845, USA; 5 Diversity Arrays Technology P/L, PO Box 7141, Yarralumla ACT 2600, Australia

## Abstract

**Background:**

Sorghum genome mapping based on DNA markers began in the early 1990s and numerous genetic linkage maps of sorghum have been published in the last decade, based initially on RFLP markers with more recent maps including AFLPs and SSRs and very recently, Diversity Array Technology (DArT) markers. It is essential to integrate the rapidly growing body of genetic linkage data produced through DArT with the multiple genetic linkage maps for sorghum generated through other marker technologies. Here, we report on the colinearity of six independent sorghum component maps and on the integration of these component maps into a single reference resource that contains commonly utilized SSRs, AFLPs, and high-throughput DArT markers.

**Results:**

The six component maps were constructed using the MultiPoint software. The lengths of the resulting maps varied between 910 and 1528 cM. The order of the 498 markers that segregated in more than one population was highly consistent between the six individual mapping data sets. The framework consensus map was constructed using a "Neighbours" approach and contained 251 integrated bridge markers on the 10 sorghum chromosomes spanning 1355.4 cM with an average density of one marker every 5.4 cM, and were used for the projection of the remaining markers. In total, the sorghum consensus map consisted of a total of 1997 markers mapped to 2029 unique loci (1190 DArT loci and 839 other loci) spanning 1603.5 cM and with an average marker density of 1 marker/0.79 cM. In addition, 35 multicopy markers were identified. On average, each chromosome on the consensus map contained 203 markers of which 58.6% were DArT markers. Non-random patterns of DNA marker distribution were observed, with some clear marker-dense regions and some marker-rare regions.

**Conclusion:**

The final consensus map has allowed us to map a larger number of markers than possible in any individual map, to obtain a more complete coverage of the sorghum genome and to fill a number of gaps on individual maps. In addition to overall general consistency of marker order across individual component maps, good agreement in overall distances between common marker pairs across the component maps used in this study was determined, using a difference ratio calculation. The obtained consensus map can be used as a reference resource for genetic studies in different genetic backgrounds, in addition to providing a framework for transferring genetic information between different marker technologies and for integrating DArT markers with other genomic resources. DArT markers represent an affordable, high throughput marker system with great utility in molecular breeding programs, especially in crops such as sorghum where SNP arrays are not publicly available.

## Background

Sorghum (*Sorghum bicolor *L.), a major staple food and fodder crop, is among the world's most important cereals, typically ranking fifth globally in terms of annual tonnage [1]. The crop is tolerant of many biotic and abiotic stresses and is often grown in more marginal cropping areas and is frequently preferentially grown in water-limited environments in both developed and developing countries [2]. In developing countries it tends to be a staple food and forage of the poor. In developed countries it is used primarily as an animal feed, and in Australia is currently grown on over 890,000 ha, producing over 2.3 M tonnes of grain [3]. More recently, tropical sorghum cultivars have garnered much attention as a cellulosic biofuels crop. Sorghum breeding programs around the world are working towards improved varieties with better quality, disease-resistance, drought tolerance and agronomic traits (e.g. [4,5]). Molecular breeding strategies are increasingly being adopted to develop genetic linkage maps and to identify genomic regions influencing traits of importance in sorghum, e.g. stay-green [6] fertility restoration [7], ergot resistance [8], midge resistance [9] and photo-period sensitivity [10,11].

Genetic linkage maps are an essential prerequisite for studying the inheritance of both qualitative and quantitative traits, to develop markers for molecular breeding, for map-based gene cloning and for comparative genomic studies. Molecular breeding is more effective if the molecular map is densely populated with markers, in order to provide more choice in the quality and type of marker and to increase the probability of polymorphic markers in important chromosomal intervals. Sorghum genome mapping based on DNA markers began in the early 1990s and numerous genetic linkage maps of sorghum have been published in the last decade [12-28]. The early maps were based primarily on RFLP markers, with more recent maps also including AFLPs and SSRs and very recently, Diversity Array technology (DArT) markers. The advent of the new DArT marker technology [29] offers a rapid and sequence-independent shortcut to medium-density whole genome scans of any plant species. As DArT assays are performed on highly parallel and automated platforms, the cost per datapoint (a few cents per marker assay) is reduced by at least an order of magnitude compared to current, gel-based technologies. Additionally, DArT clones can be readily sequenced thereby allowing marker integration into the emerging sequence of the sorghum genome . It is essential to integrate the rapidly growing body of genetic linkage data produced through DArT with the existing genetic linkage maps generated through other marker technologies. Additionally, the majority of sorghum genetic linkage maps published to date are based on crosses wider than most crosses routinely made in sorghum breeding programs. However, for application in molecular breeding strategies, genetic linkage maps based on wide crosses are often of limited utility, as they are not representative of the genome organisation and gene function of the cultivated gene pool [30]. The construction of a consensus map synthesising the information provided by multiple segregating populations, of diverse genetic backgrounds, provides a very important reference resource; it offers the opportunity to map a larger number of loci than in most single crosses, thus increasing the number of potentially useful markers across divergent genetic backgrounds and providing greater genome coverage, in addition to providing opportunities to validate marker order.

Here, we report on the comparison of the genetic linkage maps obtained from six independent component maps and on the integration of the component maps into a single consensus linkage map of sorghum. One of the component maps used, based on BTx623/IS3620C, developed at Texas A&M University and USDA-ARS scientists [25], is a reference mapping population in the sorghum genomics community and has been the subject of extensive phenotypic and genotypic analysis. Its inclusion in this study offers opportunities to link the consensus map to existing genetic and physical maps based on this population. The consensus map, consisting of over 2000 markers, also offers an opportunity to create a "bridge" between DArT and other marker systems, through the co-location of the different marker types, including RFLPs and SSRs.

## Results

### Component maps of individual populations

The parental genotypes of the six component mapping populations varied in their level of polymorphism per cross (Table 1), with the parents of the S4 population being the most diverse and the parents of the S6 population the least diverse.

**Table 1:** Summary of component mapping data used to construct the sorghum DArT consensus map

												Number of markers in common with n other populations				
Pop code	Pop pedigree	Dissimilarity Index	Generation	Pop size	Marker #	Predominant marker type	# of DArTs	# of SSRs/STSs	# of RFLPs	# morphological markers	*N *= 0	*n *= 1	*n *= 2	*n *= 3	*n *= 4	*n *= 5

TAMU-ARS	BTx623/IS3620C	0.483	RIL	137	792	DArT	303	226	259	4	493	161	101	33	3	1
S2	R890562/ICSV745	0.479	RIL	119	488	RFLP	234	10	244	0	269	87	93	34	4	1
S4	R931945-2-2/IS8525	0.639	RIL	146	410	DArT	357	51	0	2	143	130	94	38	4	1
S5	B923296/SC170-6-8	0.426	RIL	88	189	DArT	176	13	0	0	43	63	50	28	4	1
S6	BTx642/QL12	0.403	RIL	94	117	DArT	117	0	0	0	15	41	42	15	3	1
CIRAD	SAR10/SSM249	0.449	RIL	183	807	DArT	627	131	47	2	591	119	70	24	2	1

The table includes the pedigree of each population, the dissimilarity index (based on the Sokal & Michener coefficient) between the parental genotypes of each component mapping population, as calculated using the DARwin software [59], the generation and population size, and details of the number and type of markers.

**Figure 1:**
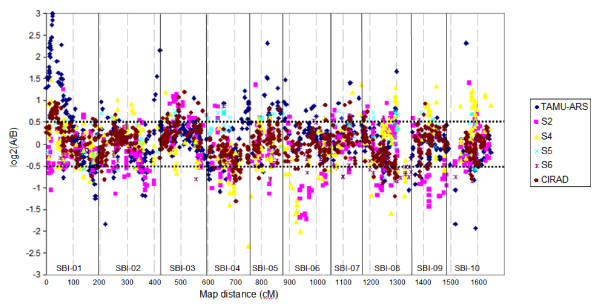
**Scatter plot representing the distribution of marker skewness of the six component sorghum maps, each dot representing one molecular marker**. Vertical solid bars distinguish the 10 chromosomes, along the total map distance (*x *axis). The *y *axis details the log2 value of the ratio of the number of individuals carrying the A allele on the number of individuals carrying the B allele. Markers outside the two horizontal dotted lines are significantly skewed as calculated by the Chi-square test.

The component maps constructed using the MultiPoint software contained between 117 (S6) and 807 (CIRAD) loci and between 88 (S5) and 183 (CIRAD) lines (Tables 1 &2, Additional File 1). The lengths of the resulting maps varied between 910 and 1528 cM (Table 2). Clusters of markers with skewed segregation were identified in all six of the sorghum linkage maps developed (Fig 1). The percentage of skewed markers was very similar across all six populations, varying from 17.1% in the CIRAD population to 24.8% in the S2 population. A significant number of the markers showing skewed segregation in each population were also linked by at least 5 cM to markers that didn't show distorted segregation patterns. For example, in the TAMU-ARS population, of the 167 markers showing segregation distortion, 51 of them were linked within 5 cM to unskewed markers. Two-thirds of these 51 markers were co-dominant marker types (SSRs or RFLPs), with the remaining one third (17 in total) markers having a dominant inheritance pattern (DArT); which reflects the relative proportion of codominant (61%) and dominant (39%) markers overall in the TAMU-ARS map. The distribution pattern of chromosomal regions associated with skewed marker segregation showed some similarity across maps, e.g. the distal end of the short arm of SBI-01 showed skewed marker segregation in four of the six maps (TAMU-ARS, S2, S4 and CIRAD); the lack of segregation distortion in the two remaining maps (S5 and S6) might be explained by poor marker resolution in this chromosomal region. It has been proposed [31] that when a chromosomal region contains four or more closely linked markers which are significantly and consistently deviating from the 1:1 ratio it can be regarded as having skewed segregation. By following this proposition and defining the closely linked markers as being less than 5 cM apart, 407 markers on the consensus map (19.8%) were identified as having skewed segregation in one or more of the component populations, covering 34% of the consensus map length. Of these 407 markers, 245 (60%) were DArT markers, which reflects the relative proportion of DArT (59%) versus non-DArT markers (41%) overall in the consensus map.

**Table 2:** Statistics of the six component maps

	TAMU-ARS	S2	S4	S5	S6	CIRAD
Number of markers	792	488	410	189	117	807
Mean marker density/cM	0.51	0.34	0.29	0.17	0.13	0.66
Map length (cM)	1528	1433	1435	1138	910	1227

The table includes the pedigree of each population, the dissimilarity index (based on the Sokal & Michener coefficient) between the parental genotypes of each component mapping population, as calculated using the DARwin software [59], the generation and population size, and details of the number and type of markers.

### Consensus map construction and features

**Figure 2:**
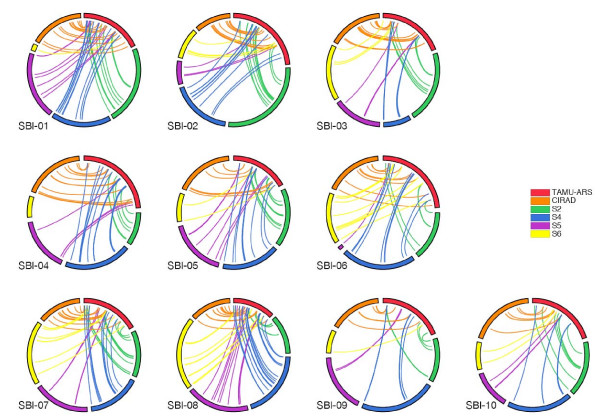
**Colinearity of locus order in component maps**. Loci that are common between pairs of populations are connected by lines. Population codes as in Table 1.

A total of 498 markers (384 of which were DArTs) were in common, i.e. they were mapped in at least two mapping populations. A total of 1557 markers (816 of which were DArTs) were unique to a particular mapping population, while seven DArT loci were mapped in five or more mapping populations. The order of those markers that segregated in more than one population was highly consistent between the six individual mapping data sets. Fig. 2 illustrates this high degree of marker colinearity of all the markers in common with the TAMU-ARS base map. A difference ratio was calculated per chromosome [27], to compare the genetic distances between each map and the TAMU-ARS base map (Table 3), where a distance ratio of 0 indicates identical genetic distances between two maps and a distance ratio of 1 indicates complete dissimilarity of genetic distances between two maps. The number of intervals in common with the TAMU-ARS population varied across populations, from just 32 in the S6 map to 113 in the CIRAD map. The overall difference ratios in genetic distance between the TAMU-ARS map and the five other maps varied from 0.0045 (S4) to 0.125 (S5). The difference ratios also varied for each chromosome, with SBI-04 having the lowest difference ratios (an average of 0.09) and SBI-02 having the highest difference ratios (an average of 0.25). The high difference ratios observed across maps for some chromosomes, specifically SBI-02, SBI-03, SBI-05, SBI-09 and SBI-10, were due to the low number of sampling intervals; in some cases as low as 1 interval in common per LG (e.g. S6/TAMU-ARS SBI-05 and SBI-10). In some cases, there were no intervals in common for particular chromosomes across maps, e.g. S6/TAMU-ARS for SBI-01, SBI-04 and SBI-09 and S5/TAMU-ARS for SBI-06.

**Table 3:** The difference ratio of genetic distance in the common marker intervals between the TAMU-ARS map and the five other component maps

	S2	S4	S5	S6	CIRAD
# intervals in common with TAMU-ARS	92	99	46	32	113
Difference ratio	0.0609	0.0045	0.1248	0.0174	0.0469

As observed previously [32], markers mapping to more than one locus can create problems during consensus map construction, if not recognised. In the present study, just under one quarter (24.2%) of the total number of unique markers mapped across the six component maps were in common in more than one population, and of these only 35 mapped to two different loci in different populations (Additional File 2). As expected, due to the use of the same DArT array across populations, the majority of the markers in common across maps were DArT markers (77.7%). Consequently, a higher proportion of the multicopy markers overall were DArT markers (31) versus non-DArT markers (4); 3 SSRs (gap42, txp25 and txp265) and 1 RFLP (txs443). SBI-02 contained the highest number of multicopy markers (13).

**Figure 3:**
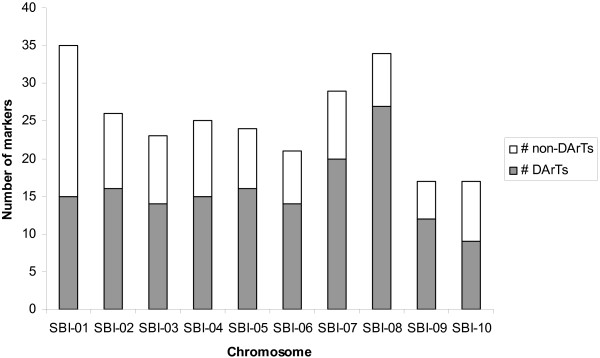
**Total number and proportion of DArT vs non-DArT markers used as bridge markers on the consensus map, per chromosome**.

The sorghum consensus map consisted of a total of 1997 markers mapped to 2029 unique loci (1190 DArT loci and 839 other loci; full details available in Additional File 3). Of the 1997 unique markers placed on the consensus map, there were 493 (24.7%) common markers; only 5 common markers from the total of 498 across all six component maps were excluded from the consensus map due to inconsistency in marker location. Of these common markers included, 251 were selected as bridge markers on the 10 sorghum chromosomes, i.e. markers which mapped to the base map (TAMU-ARS) and which were also present and in a consistent location in one or more of the other mapping populations. SBI-01 had the highest number of bridge markers overall (35), of which 42.8% (15) were DArT markers (Fig. 3). SBI-09 (17) and SBI-10 (17) had the lowest number of bridge markers, of which 70.5% and 52.9% were DArT markers, respectively. The remaining chromosomes had between 34 (SBI-08) and 21 (SBI-06) bridge markers, of which between 79.5% (SBI-08) to 60.0% (SBI-04) were DArT markers. The 251 bridge markers, which consisted of 158 DArT markers, 64 SSR markers and 29 RFLP markers, spanning 1355.4 cM with an average density of one marker every 5.4 cM, were used for the projection of the remaining markers onto the bridge consensus map.

On average, each chromosome on the consensus map contained 203 markers of which 58.6% were DArT markers. Chromosome SBI-01 had the highest number of markers (294) overall but one of the lowest percentages of DArT markers (49.7%) (Table 4), with an average marker density of 1/0.65 cM, followed by chromosome SBI-02 (270 in total, 54.8% DArTs), with an average marker density of 1/0.85 cM. Chromosome SBI-07 had the lowest number of markers (129, 57.4% DArTs).

**Table 4:** Summary of markers per chromosome integrated into the sorghum DArT consensus map

LG	# Bridge	# DArTs (%)	# non-DArTs (%)	Total	Multicopy	Length (cM)	Marker density
SBI-01	35	146 (49.7%)	148 (50.3%)	294	8	191.8	0.65
SBI-02	26	148 (54.8%)	122 (45.2%)	270	13	229.6	0.85
SBI-03	23	105 (44.5%)	131 (55.5%)	236	3	172.3	0.73
SBI-04	25	144 (70.2%)	61 (29.8%)	205	7	169.4	0.83
SBI-05	24	122 (69.7%)	53 (30.3%)	175	9	118.5	0.68
SBI-06	21	105 (61.5%)	68 (39.5%)	172	2	166.4	0.97
SBI-07	29	74 (57.4%)	55 (42.6%)	129	9	132.8	1.03
SBI-08	34	131 (69.3%)	58 (30.7%)	189	7	131.9	0.69
SBI-09	17	108 (62.4%)	66 (37.6%)	174	2	175.6	1.01
SBI-10	17	107 (57.8%)	78 (42.2%)	185	10	115.2	0.62

***Totals***	***251***	***1190***	***839***	***2029***	***70***	***1603.5***	***Mean = 0.79***

**Figure 4:**
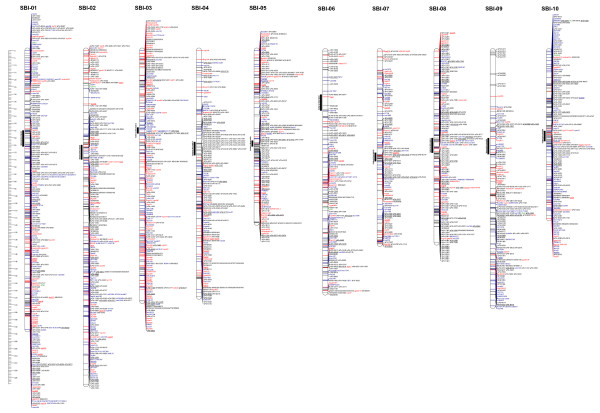
**A consensus map of sorghum derived from six component maps**. Marker type is indicated by colour; DArT (black), SSR/STS (red), RFLP (green) and gene (blue). Bridge markers are underlined; attached markers are in italics and multicopy markers have an * suffix. The bar on the left hand side shows the distance in centiMorgans from the top of each chromosome. Heterochromatic regions are indicated by a bar to the left of each chromosome.

The consensus map spanned a total length of 1603.5 cM, based on the distances calculated from the TAMU-ARS segregation data. Chromosome sizes ranged from 229.6 cM (SBI-02) to 118.5 cM (SBI-05) (Fig. 4). The 'sPb' DArT markers alone spanned 97.7% of the total length of the consensus map, ranging from 100% of chromosomes SBI-02, SBI-03, SBI-05 and SBI-06 to 94.1% coverage for SBI-01 and SBI-09.

The approximate locations of the pericentromeric regions of heterochromatin were identified (Fig. 4), based on the integration of sorghum linkage, cytogenetic and physical maps [33]. Non-random patterns of DNA marker distribution were observed, with some clear marker-dense regions and some marker-rare regions. The consensus map had only 3 gaps larger than 10 cM and only 9 gaps between 7 and 10 cM; the longest one (13.4 cM) on the distal end of the long arm of SBI-05; one (10.9 cM) on the distal end of the long arm of SBI-08, and one (13 cM) on the distal end of the short arm of SBI-09. On most chromosomes, at least one significant concentration of loci appeared to correspond to the centromeric region (also observed in [26]), e.g. 35 markers co-segregated around the centromeric region of SBI-04 and 33 markers co-segregated around the centromeric region of SBI-08. The proportion of DArT markers in the centromeric regions ranges from 36.3% on SBI-02 to 100% on SBI-04, with an average of 64.4% across all chromosomes, which reflects the overall proportion of DArT markers to non-DArT markers on the map.

## Discussion

The final consensus map comprised 2029 loci, spanning 1603.5 cM, following the integration of 6 individual maps derived from 6 distinct RIL mapping populations. It has allowed us to map a larger number of markers than possible in any individual map, to obtain a more complete coverage of the sorghum genome and to fill a number of gaps on individual maps. Only two other published sorghum genetic linkage maps are of a comparable marker density; the BTx623/IS3620C map consisting of 2926 loci spanning 1713 cM [25] and the BTx623/*S. propinquum *map consisting of 2512 loci spanning 1059.2 cM [26]. While both of these previously published maps have a higher overall marker density than the present DArT consensus map; 1 marker/0.42 cM [26], 1 marker/0.59 cM [25] vs. 1 marker/0.79 cM in the presented consensus map, these maps are based on high numbers of RFLP markers [26] or AFLP markers [25] and it can be argued that the sequential nature of gel-based marker systems such as RFLPs and AFLPs involves high costs and is more labour intensive per assay thus DArT markers may represent the most suitable markers for molecular breeding strategies. DArT markers, with their high multiplexing level (all the DArT markers reported here were analysed in a single assay per population), offer sorghum breeding programs an alternative and low-cost approach to whole-genome profiling and the final consensus map presented here consists predominantly of DArT markers (1190; 59%), in addition to 839 non-DArT markers (497 RFLPs, 334 SSRs or STSs and 8 morphological markers).

The overall consensus map marker order was in good agreement across the individual maps. Locally, the consensus map resolution was slightly compromised by occasional inconsistencies in groups of markers, commonly covering about 1–6 cM, but also swaps of individual markers over even longer distances. The majority of the 77 observed marker order inconsistencies involved closely-spaced markers. Inversion is a common feature of closely spaced markers and this phenomenon has been observed previously in sorghum when aligning different sorghum maps [27,30]. These marker order rearrangements could be real, they could be due to error in one of the small mapping populations or they could be explained by the statistical uncertainty of orders at the cM-scale that is inherent in datasets derived from a limited number of RILs. Of the 498 markers in common across all 6 maps, in only 5 cases did markers map to a truly incongruous location on the corresponding linkage groups in alternative populations, which could be explained by mapping paralogous loci in different populations. A similar 1% frequency of paralogous loci was recently observed by [30] when aligning genetic linkage maps derived from both inter- and intra-specific sorghum populations. Such marker ordering inconsistencies are frequently observed for consensus maps and can be related to the overall number and distribution of commonly mapped bridge markers used for building the framework of the consensus map. For constructing the present DArT consensus map, 251 markers were used as bridge markers (12.5% overall) spaced at average intervals of 5.4 cM. This bridge marker frequency is comparable to other recent consensus map studies, including [34] who used 10% of all markers as bridge markers to construct a consensus map for barley from 3 doubled haploid populations.

Differences of local recombination frequencies (map length) between populations can also effect marker ordering between maps, and the importance of similar recombination frequencies across individual maps when constructing a consensus map has previously been noted [35]. A difference ratio was therefore calculated per chromosome, derived from the equation for the distance measurement of interval variables [36] by [27], to compare the genetic distances on each map with the TAMU-ARS base map. The overall difference ratios in genetic distance between the TAMU-ARS map and the five other maps were low and varied from 0.0045 (S4) to 0.12 (S5) and were comparable with a recent study [27] that calculated a difference ratio of 0.05 between two sorghum maps. The low difference ratios observed indicate that there is good agreement in overall distances between common marker pairs across the component maps used in this study. It also provides justification for the "neighbours" consensus map construction strategy adopted here and the use of the TAMU-ARS genetic distances for the locus positions of the bridge markers along each chromosome. It can also be argued that map distance estimates are less important than marker order, as map distances do vary between different genetic linkage maps by several centimorgans [37], and that the marker order is the most critical feature for further application of the map, for example, for map-based cloning. Additionally, the synthetic approach to consensus map development, based on the integration of separately constructed component maps, was recently reported to be the preferable consensus map construction strategy, compared to building a consensus map *de novo *from an integrated set of segregation data [32], at least until improved or alternative software options become available.

### Consensus map features

The non-random distribution of markers across the consensus map, due to both clusters and gaps of markers across chromosomes, is a feature that has also been observed in previous sorghum maps. Figure 4 indicates that there is a clustering of markers around the centromere for every chromosome, with the exception of SBI-06. Such marker-dense regions around the centromeres were also observed by [26]. This is also supported by the recent observation by [33] that the pericentromeric heterochromatic regions of sorghum chromosomes showed much lower rates of recombination (~8.7 Mbp/cM) compared to euchromatic regions (~0.25 Mbp/cM), with the average rate of recombination across the heterochromatic portion of the sorghum genome being ~34-fold lower than recombination in the euchromatic region. Similarly, the sparseness of markers on the short arm of SBI-06 could also be explained by the observations of [33] that this chromosome arm showed a relatively low rate of recombination compared to other regions of euchromatin (~2.3 Mbp/cM vs. the overall average of ~0.25 Mbp/cM). Both DArT and non-DArT markers clustered around the centromeres, however a slightly higher overall proportion of DArT markers (71% of all markers in the centromeric regions) in these regions were observed. This is in contrast to the recent high-density DArT consensus map developed for barley, which [32] found that DArT markers were significantly less clustered at most centromeric regions of barley chromosomes compared to non-DArT markers. Marker redundancy can also enhance the non-random marker distribution pattern. In previous studies [32,38,39], a low level of DArT marker redundancy has been observed, however during the process of consolidating the most informative DArT clones in new arrays, the large majority of redundant markers are excluded from the final DArT array, and hence DArT marker redundancy should be minimised.

In addition to the uneven distribution of recombination events along chromosomes and the potential for the confounding effects of marker redundancy, non-random marker distribution can also be due to the preferential survey of DNA polymorphism that is unevenly distributed along chromosomes. In particular, areas of low marker density may correspond to regions of similar ancestry or identity by descent in the germplasm included in the initial diversity representation for the development of the sorghum DArT markers [28]. In the present DArT consensus maps, there were 3 gaps larger than 10 cM; one on the distal end of the long arm of SBI-05, one on the distal end of the long arm of SBI-08 and one on the distal end of the short arm of SBI-09. These regions of low marker density may therefore be associated with genomic regions that were identical by descent or that had very limited genetic variability in the initial diversity representation used for the development of the DArT array. An alternative hypothesis is that because, in total, nine of the twelve parental genotypes of the six mapping populations used in this study were included on the initial diversity representation, the gaps could be a true reflection of co-ancestral regions between the parents, as opposed to a result of the composition of the array, and maybe suggestive of genomic regions containing key adaptive genes which have been fixed through selection through the pedigree. Regions of low marker density have been observed previously; even on the densest meiotic linkage map produced yet, for potato [40], a gap spanning 14 recombination units was observed. The authors [40] postulate that this could be due either to recombination hot spots or could also indicate fixation (homozygosity) of the potato genome in this region. Non-random marker distribution can also be associated with other interesting features of sorghum genome organisation. It has also been noted [26] that sorghum chromosomes have cytologically distinguishable knobs, which may account for some marker excesses or deficiencies.

Approximately 75% of the consensus map (524 markers spanning 1495 cM) was associated with markers which had skewed segregation in one or more of the six component maps. However, only 407 (19.8% of the markers on the consensus map) of the 524 skewed markers were linked by less than 5 cM to other markers showing distortion. The 117 markers with skewed segregation that were linked by at least 5 cM to markers that weren't distorted could reflect residual levels of heterozygosity in the lines (when scored with dominant markers), due to either natural or artificial selection, sampling bias due to lower numbers of markers in these regions or mis-scoring of the markers. Skewed segregation was observed for both DArT and non-DArT markers; no one marker type showed a particular tendency for skewness. Marked differences were observed, however, for the distribution of markers with skewed segregation across chromosomes, although there was some similarity between the component maps, e.g. the short arm of SBI-01 showed skewed marker segregation in four of the six maps (TAMU-ARS, S2, S4 and CIRAD). Highly significant deviation from the expected 1:1 segregation ratio on SBI-01 towards the BTx623 allele was also observed by [25], which affected almost the entire linkage group. The authors [25] also noted other reports of similar skewed segregation in the same genomic region and observe that strong and consistent segregation distortion in one genomic region is less likely to be due to sampling error and more likely suggests selection favouring one parental allele. On the DArT consensus map, SBI-01 has the highest proportion of chromosomal regions associated with skewed segregation (67%). Two other chromosomes (SBI-04 and SBI-08) also have over 50% of the chromosomal regions associated with skewed segregation (51.6% and 54.1%, respectively), once again also observed by [25]. SBI-07 has a significantly lower portion of the chromosome associated with skewed segregation (9.6%) than any other chromosome on the consensus map. This non-random and consistent distribution pattern of skewed segregation lends weight to previous proposals [18,25,40,41] that distorted segregation is due to the elimination of gametes or zygotes by a lethal factor located in a neighbouring region of the marker. Higher frequencies of skewed markers have also been observed in RIL populations, compared to doubled haploid, backcross or F_2 _population structures [31], due to increased opportunities for selection across generations; all six component maps in the current study are based on RIL populations.

Of the 1997 markers included in the DArT consensus map, 35 mapped to different chromosomes in the component maps. The frequency of multicopy markers detected in this study (1.8%) is much lower than observed by [26], who found that 17% of RFLP probes mapped to multiple locations. This could be explained by the differences in marker types. It has been found that DArTs, as a hybridisation-based bi-allelic marker, inherently select against multi-locus markers [32], as the hybridisation intensities measured for such multi-locus markers tend to appear monomorphic. Variation in the frequency of multicopy markers was observed across chromosomes, with SBI-07, SBI-10, SBI-02 and SBI-05 having a multicopy marker frequency greater than 5%. SBI-06 had the lowest multicopy marker frequency (1.1%). A tendency for the multicopy markers to be present in the centromeric regions across chromosomes was also observed, with approximately 22% of all multicopy markers occurring in the pericentromeric heterochromatic regions, whilst overall only 13% of all markers included in the consensus map are located in the centromeric regions. Centromeric suppression of recombination is associated with the accumulation of repeated sequences [33] and could explain the tendency towards marker duplication. The non-random distribution of multicopy loci across chromosome pairs has been reported previously [20,26]. It has been observed [26] that the duplication of sorghum chromatin closely resembles the pattern for rice, showing ancient duplications in some regions. However, very little evidence was found in the current study for co-linearity between chromosomes, lending weight to the argument against an ancient polyploidisation event in the evolution of the sorghum genome [42-44]. It has also been previously observed [26] that 30% of the sorghum genome showed correspondence to two or more unlinked intervals which the authors postulated could either be due to very localised colinearity or which may reflect more recent duplications superimposed on more ancient ones.

### Utility of the consensus map for genomics and breeding applications

The DArT consensus map presented in this paper will help link information on sorghum diversity and QTLs to the sorghum physical map and to the sorghum genome sequence. The availability of the primer sequence information for the majority of SSRs  and probe sequence information for a subset of RFLP markers with the prefixes *bcd*, *bnl*, *cdo*, *csu*, *psb*, RG, *rz *and *umc * included on the consensus map already provides immediate opportunities to anchor the presented consensus map to the physical map, hence faciliating sequence mapping of known genes from other species, taking advantage of known syntenic relationships between sorghum, rice, maize and other grasses [45,46], in addition to a positional cloning approach to identify candidate genes underlying QTLs flanked by sequenced mapped SSRs or RFLPs. To demonstrate this, 42 RFLPs included on the consensus map were sequence mapped on the rice genome (TIGR; ) and bin-mapped on the maize genome (MaizeGDB; ); data presented in Additional File 4. The syntenic genomic regions between sorghum, rice and maize were largely as expected, at the macro-level [45,46]. With the recent availability of both the rice and sorghum whole genome sequences, and the on-going sequencing of the maize genome, however, not only the macro-level synteny, but genic microsynteny can now be furthered explored. As an example, comparisons for fifteen predicted genes (downloaded from ) in the 265,271 bp euchromatic region between the two RFLP markers *rz*630 and *umc*90 on the sorghum genome (SBI-01) were made between rice and sorghum. BLAST similarity between the sorghum predicted genes and the rice sequence, requiring hits with E ≤ 1e-10 based on BLASTn, are detailed in Additional File 5. Over 73% conserved synteny among the 15 predicted genes was observed; comparable to microsyntenic levels (72%) observed previously [46] in euchromatic genomic regions in rice and sorghum. Far greater microcolinearity has also been observed [46] in euchromatic regions, compared to heterochromatic regions. Further detailed evaluation of the level of genic microcolinearity, both in euchromatic and heterochromatic regions, between rice and sorghum based on the whole genome sequence analysis will provide invaluable knowledge for cereal scientists and will provide new opportunities for sorghum researchers to link QTL and gene information aligned to genetic linkage maps directly to the whole genome sequence and predicted genes. The on-going sequencing of the sorghum DArT clones, when integrated with the whole genome sequence, offers many opportunities to greatly accelerate gene discovery and analysis in addition to the opportunity to convert the recombination fractions on the consensus map to physical map distances (cM to kb), affording new prospects for the progress of genomic applications. The sorghum whole genome and DArT clone sequences can also be exploited for targeted marker development for specific genomic regions. Because of ease of sequence analysis, DArT markers have a significant advantage over AFLPs for positional cloning efforts due to the difficulty in sequencing AFLPs that, therefore, cannot be readily integrated into the whole genome sequence.

An additional use of the presented DArT consensus map is in whole genome profiling-assisted breeding. The marker density on the consensus map is sufficient to provide a better choice of markers for specific breeding populations to ensure adequate polymorphic marker coverage in regions of interest. Further, the marker density on the consensus map is suitable for whole genome pedigree analysis, and calculating identity-by-descent through generations. The consensus map provides a large number of markers along the length of the chromosome that can be used to genotype individuals for detecting recombinants, fixing loci, restoring a recurrent genetic background, or assembling complex genotypes in complex crosses. The co-location of a range of marker types (DArTs, RFLPs and SSR markers) on the consensus map will enable sorghum breeders to quickly identify target loci through whole-genome DArT scans and then select markers of interest from the same region for marker-assisted selection.

## Conclusion

The integration of six distinct genetic maps into a consensus map has made it possible to obtain a general order and distances for a greater number of markers, and to obtain more complete coverage of the sorghum genome. The consensus map presented here is a good estimation of the marker position from the six component maps. The exact fine marker order may differ slightly in other populations, and users should be prepared to establish the order for closely linked markers in their mapping and breeding populations. The obtained consensus map can be used as a reference map to develop genetic studies in different genetic backgrounds, in addition to providing a framework for transferring genetic information between different marker technologies and for integrating DArT markers with other genomic resources.

## Methods

### Mapping populations

A total of six component mapping populations were used to integrate over 2000 unique loci, including 1182 unique DArT markers, into a single consensus map (Table 1). The TAMU-ARS population, developed at Texas A&M University, is a reference mapping population and has been subject to extensive phenotypic and genotypic analysis [14,20,22,23,25]. One of the TAMU-ARS population parents, BTx623, is the genotype selected for the sorghum genome sequencing project [47]. The four mapping populations, S2, S4, S5 & S6, were developed at the Department of Primary Industries & Fisheries, Queensland by D. Jordan (pers. comm.) and have also been used in studies to map target traits (e.g. [9,28,48]). The CIRAD population was developed at the Saria Research Station, Burkina Faso by Trouche (pers. comm.), from the cross between the genotype SSM249 (guinea from Burkina Faso) and the genotype SARIASO10 (caudatum from Burkina Faso) and has been used for QTL mapping on target traits (Rami, pers. comm.).

### Genotyping data

Several sources of markers, including DArTs, RFLPs and SSRs, mapped in the individual component maps were used to prepare the sorghum consensus map. Segregation data from a total of 331 unique SSRs/STSs (with prefix: *cup *as described by [49]; *gap *and Sb as described by [50] and [22]; gpsb, msbcir and SSmsbcir as described by CIRAD (Rami, pers. comm.); SbAG as described by [51] and *txp *as described by [22,23] and 497 unique RFLPs (from barley cDNA with *bcd *prefix; from maize genomic and cDNA probes with prefix: *bnl*, *csu*, *isu *and *umc*; from oat cDNA with *cdo *prefix, from sorghum genomic DNA with *psb *and *txs *prefix, from rice genomic and cDNA probes with RG and *rz *prefix, and from sugar cane genomic and cDNA probes with, EST, FC, GE, JH, MT, RG, SSCIR, SG, ST and STr prefixes, as described by [9,18,20,26]) across the six component mapping populations were included in this study. All six populations were genotyped with an identical set of DArT markers from a *Pst*I+*Ban*II representation ('sPb' markers), following the methodology detailed in [28]. The CIRAD population was also assayed with a unique set of MITE-DArT markers (Bouchet, pers. comm.). The segregation data of 489 non-DArT marker loci mapped in TAMU-ARS were obtained from P. E. Klein (pers. comm.) and integrated with 306 polymorphic DArT markers. The 2454 AFLP loci mapped in the TAMU-ARS population by [25] were excluded from this study due to the problems in transferability of this marker type among laboratories, as discussed by [52]. Marker data previously generated for the four DPI&F mapping populations (S2, S4, S5 and S6) were integrated with segregation data from a total of 884 DArT markers. The non-DArT data for the S4 population consisted of both SSRs and AFLPs [46], however as with the TAMU-ARS data set, the AFLP markers were excluded from this study. The non-DArT data sets previously generated for the S2, S5 and S6 populations are unpublished (Jordan, pers comm.). For the CIRAD map, segregation data for 180 non-DArT loci, obtained from J.F. Rami (pers. comm.), were integrated with segregation data from a total of 627 DArT markers, which included 269 newly identified polymorphic MITE DArT clones. With the exception of DPI&F mapping population S2, the component maps' segregation data predominantly consisted of DArT markers. DArT markers with a quality parameter and a call rate both greater than 77% were selected for inclusion in the component genetic linkage maps. DArT markers with a quality parameter between 75 and 77% were incorporated on a case-by-case basis.

### Marker nomenclature

DArT marker names are standardised and automatically generated by a DArT-specific Laboratory Information Management System (DArTdb; DArT P/L, Canberra, Australia). Different laboratories used slightly different names for the same SSR and RFLP markers. Non-DArT marker names were therefore curated to the extent required to create an unambiguous nomenclature.

### Component genetic linkage map construction

The component genetic linkage maps of the six sorghum mapping populations were constructed using MultiPoint software [53]. The RIL_Selfing population setting was selected and a maximum threshold rf_s _value of between 0.1 to 0.40 was used to initially group the markers into a minimum of ten linkage groups. Multipoint linkage analysis of loci within each LG was then performed and marker order was further verified through re-sampling for quality control via jack-knifing [54]. Markers that could be ordered with a jack-knife value of 90% or greater were included as 'framework' markers, with any remaining markers causing unstable neighborhoods being initially excluded from the map, including redundant markers mapping to the same location. Following a repeated multipoint linkage analysis with the reduced set of markers for each LG to achieve a stabilised neighbourhood, the previously excluded markers were attached by assigning them to the best intervals on the framework map. Finally, known chromosomal locations of a subset of the DArT [28], SSR and RFLP [25] markers were used to assign the linkage groups to sorghum chromosomes, SBI-01 to SBI-10 according to the recent nomenclature system as suggested by [55]. The Kosambi [56] mapping function was used to calculate the centimorgan (cM) values. The marker orders generated by MultiPoint for each component map were then displayed in map order per LG as color-coded graphical genotypes in Microsoft Excel using a conditional cell formatting formula. The graphical genotypes of these maps were then investigated to identify 'singletons' (apparent double crossover events) pointing to either a potentially incorrect marker order or a genotyping error. Individual singletons were not, however, replaced with missing data, in contrast to [57]. The observation of singletons depends on their context of flanking markers and also the population type; the number of recombination events that can have occurred in a RIL population make it more likely that a singleton represents a real event compared to a DH population, which has only had one generation of cross-overs.

The distance measurement of interval variables between 2 individuals, proposed originally by [36] and modified by [27] was used to compare the genetic distances between each map and the TAMU-ARS base map. The modified distance measure [27] is based on the following formula:

$$\text{Difference ratio}=\frac{\sum \left|{\text{A}}_{ik}-{\text{B}}_{ik}\right|}{{\text{A}}_{i}+{\text{B}}_{i}}$$

where A_*ik *_is the length (cM) of the *k*th shared marker interval on the *i*th chromosome of map A, and B_*ik *_is the length (cM) of the *k*th shared marker interval on the *i*th chromosome of map B. The Σ|A_*ik *_- B_*ik*_| is the absolute value of the length difference of each shared marker interval on the *i*th chromosome between maps A and B, and A_*i *_+ B_*i *_is an additive value of all shared intervals for the *i*th chromosome of maps A and B which is used to normalise the difference value, Σ|A_*ik *_- B_*ik*_| [27].

### Construction of the consensus map

The locus positions from the six component maps were merged to build a 'synthetic' map using basic Microsoft Excel functionalities. This strategy differs from the alternative approach of constructing a consensus map using the segregation data from different mapping populations to compute the optimum order of loci [32]. The TAMU-ARS map was selected as the 'base' or reference map, as the one containing the largest number of common loci across populations and the one with the greatest genome coverage. Bridge markers were initially identified as having an identical name and being present in TAMU-ARS and at least one of the other 5 mapping populations and having a similar map position in the different mapping populations concerned. Markers with the same name that had inconsistent positions in different populations were not considered as bridge markers. The TAMU-ARS distances were used for the locus positions of the bridge markers along each chromosome. This framework map then served as a backbone onto which the remaining loci from each component map were projected, in a "neighbours" map approach as described by [58]. For a target locus, the two nearest flanking bridge markers shared by the framework map and by the component map were identified and the coordinate of this locus was calculated relative to the ratio of the intervals defined by the flanking bridge markers on the two maps. For placing markers at group extremities, projection was based on the relative genetic distance of common markers nearest to the end of the LG between the framework map and the component map.

## Authors' contributions

ESM carried out the mapping analyses and drafted the manuscript; JFR was involved in the development of the CIRAD population, contributed to the mapping analyses, and editing of the manuscript; SB was involved in the generation of DArT data for the CIRAD mapping population and participated in the mapping analysis; PEK was involved in the development of the TAMU-ARS mapping population, generation of non-DArT marker data for the TAMU-ARS population and editing of the manuscript; RRK was also involved in the development of the TAMU-ARS mapping population, generation of non-DArT marker data for the TAMU-ARS population and editing of the manuscript; AK supervised the generation of DArT data for all mapping populations, participated in the study's design and helped to draft the manuscript; PW contributed to quality assessment of sorghum clones and data generated for this study and editing of the manuscript; LX was involved in the generation of DArT data for all mapping populations; KH was involved in the generation of marker data for the DPI&F mapping populations; DRJ conceived of the study, participated in its design and coordination, mapping analyses and helped to draft the manuscript.

## Supplementary Material

Additional file 1 **Locus positions in the component maps.** Excel spreadsheet containing a list of all component map loci, the chromosome and position of each locus. Click here for file

Additional file 2 **Multicopy marker summary.** Excel spreadsheet containing details of the total numbers of multicopy markers identified per chromosome on the sorghum DArT consensus map Click here for file

Additional file 3 **Locus positions in the consensus map.** Excel spreadsheet containing a list of all consensus map loci and their features; data include the chromosome and position of each locus, details of which component map each locus was mapped in, and multicopy marker details. Click here for file

Additional file 4 **Comparison of 42 RFLP markers across sorghum, rice and maize.** Excel spreadsheet containing a list of 42 RFLP markers, and their physical location on both the sorghum and rice genome and chromosome and bin identity in maize. Click here for file

Additional file 5 **Sorghum/rice gene microcolinearity across a 0.26 MB region of SBI-01 between rz630 and umc90.** Excel spreadsheet containing a list of 15 predicted sorghum genes on SBI-01, between rz630 and umc90, their physical location and predicted function, and the homologous rice gene ID, physical location and predicted function of the best hit based on BLASTn and the total number of hits at E = 1e-10. Click here for file
